# Ultra-high-resolution imaging of intracranial flow diverters with photon counting CT: A comparative phantom study with flat-panel CT

**DOI:** 10.1038/s41598-025-12713-0

**Published:** 2025-07-21

**Authors:** Christoph Johannes Maurer, Ansgar Berlis, Dmitrij Pinekenstein, Michael Wolf, Gebhard Östreicher, Lars Behrens, Franz Josef Stangl

**Affiliations:** 1 Department of Diagnostic and Interventional Neuroradiology, University Hospital Augsburg, Stenglinstraße 2, 86156 Augsburg, Germany; 2 Faculty of Medicine, Martin Luther University Halle-Wittenberg, Dorothea- Erxleben-Lernzentrum, Halle, Germany; 3 Medical Physics and Radiation Protection, University Hospital Augsburg, Augsburg, Germany

**Keywords:** Artifacts, Flow diverter, Flat-panel CT, Image quality, Ultra-high-resolution imaging, Neuroscience, Neuro-vascular interactions, Neurovascular disorders

## Abstract

Flow diverters are a crucial element in the treatment of intracranial aneurysms. However, the optimal non-invasive follow-up imaging modality, particularly for the detection of in-stent stenosis, remains uncertain. This study aims to compare the performance of photon-counting detector CT (PCD-CT) in ultra-high-resolution (UHR) mode with flat-panel CT (FP-CT) for the evaluation of intracranial flow diverters. A phantom model for intracranial vessels was used to evaluate 15 flow diverters of various sizes and designs. Imaging was performed using both PCD-CT and FP-CT. Qualitative assessment of the stent lumen was conducted by three experienced neuroradiologists using a 5-point Likert scale. Quantitative analysis included measurements of lumen area, contrast to noise ratio and signal to noise ratio. FP-CT provided a significantly larger assessable stent lumen than PCD-CT at all dose levels (p < 0.05), with no significant differences between PCD-CT dose levels (p = 0.999). Increasing PCD-CT dose did not improve lumen visualization. SNR and CNR increased with PCD-CT dose (p < 0.001), peaking at CTDI 20, but showed diminishing returns beyond CTDI 10. Flow diverter diameter correlated positively with SNR and CNR (p < 0.05). Subjective image quality improved with PCD-CT dose (p < 0.001) but showed no significant difference beyond 10 mGy (p > 0.05). FRED devices had the lowest ratings, independent of imaging modality (p = 0.80). Our study demonstrated that while FP-CT provided superior visualization of the flow diverter lumen in a head phantom vessel model, subjective assessability ratings were comparable between FP-CT and PCD-CT when evaluated by experienced readers. PCD-CT at a CTDIvol of 10 mGy offered the best balance between image quality and radiation dose, making it a viable alternative for post-interventional assessment of flow diverters.

## Background

Since their introduction in clinical aneurysm treatment in 2008^5,11^, flow diverters have become an indispensable tool in the arsenal of endovascular neurointerventionalists worldwide for both ruptured and unruptured aneurysms^6,16,22^. However, follow-up imaging is crucial, as a significant number of patients develop in-stent stenosis during the process of vessel remodeling caused by the implanted flow diverter^17,28^. In-stent stenosis due to intimal hyperplasia is typically diagnosed during angiographic follow-up. Aside from digital subtraction angiography, flat-panel CT (FP-CT) with intravenous contrast media is increasingly employed due to its non-invasive nature^4,8,10,21,23^.

With its improved detector properties, the recently introduced photon-counting detector CT (PCD-CT) has been proposed as an alternative for follow-up imaging of intracranial flow diverters to evaluate stent patency, adherence of the flow diverter to the vessel wall and intima hyperplasia^1,7,12,27^.

PCD-CT uses direct conversion of X-ray photons into electrical signals, eliminating the need for septa between detector elements. This allows for smaller detector pixels and improved spatial resolution without loss of dose efficiency, as there are no inactive regions between pixels^13^.

Therefore, imaging in instances where high resolution is imperative, such as in the monitoring of flow diverters, can be accomplished through the utilization of the clinically accessible ultra-high-resolution (UHR) mode. Additionally, reduced artifacts and image noise in PCD-CT may contribute to an improved evaluation of the implanted devices^12,26^. Depending on the specific clinical requirements, PCD-CT may enable simultaneous improvements in image quality and dose reduction, or achieve better image quality with a higher dose. However, a direct comparison for flow diverter evaluation between these two imaging modalities with clinically available protocols is still lacking.

The objective of this study was to assess the performance of PCD-CT in UHR mode in comparison to FP- CT for imaging flow diverters in a vessel phantom.

## Results

### Visible stent lumen

**Fig. 1:**
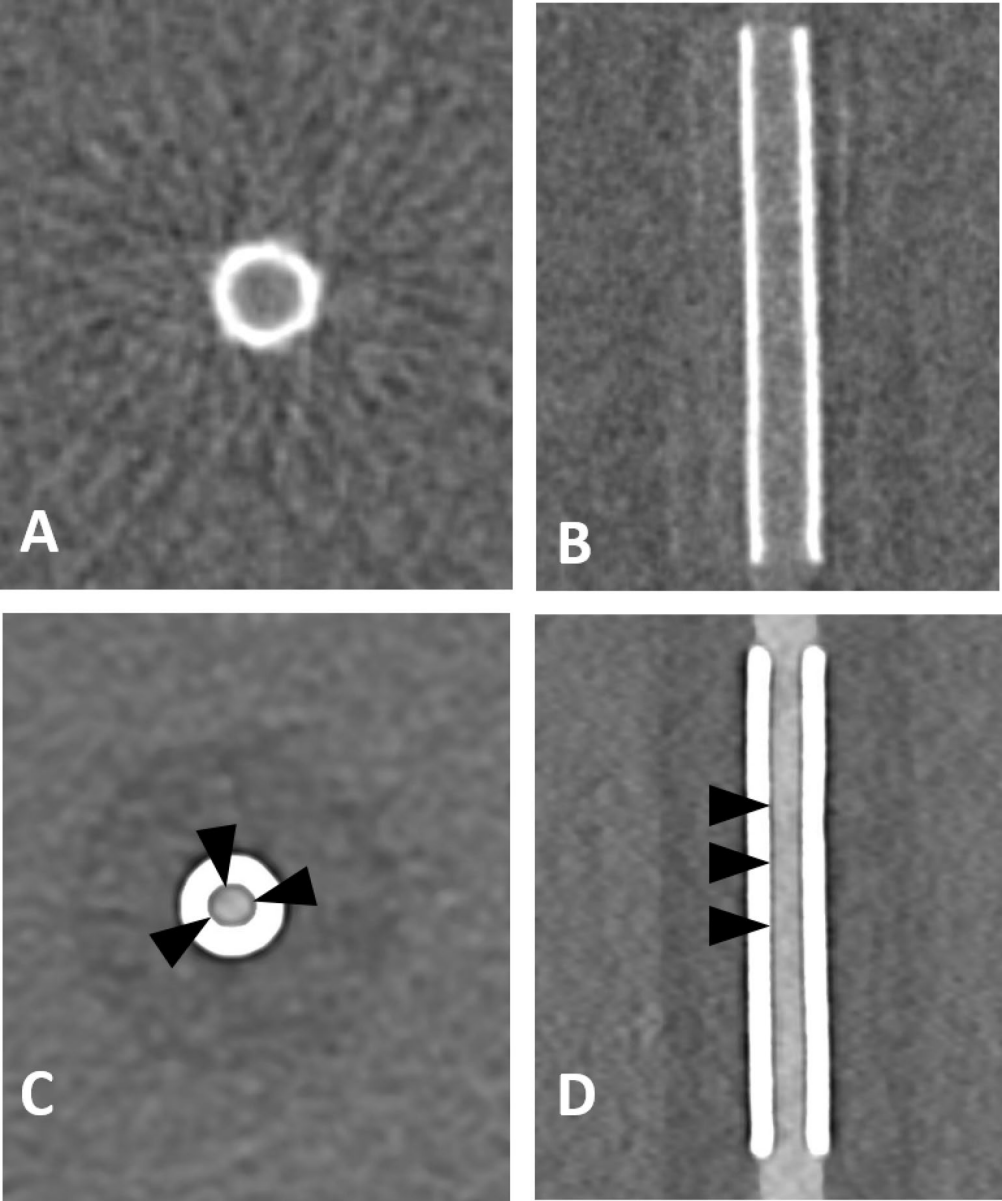
Flat panel CT (**A**, **B**) and photon-counting detector CT (CTDIvol 20 mGy; **C**, **D**) with axial and sagittal reconstructions of a Silk Vista 2.5 × 15 mm flow diverter. Compared to FP-CT, PCD-CT shows a smaller apparent lumen and a hypodense artifact adjacent to the stent wall (arrowheads). Window settings were manually optimized for contrast: A, B) W: 700 HU, L: 2000 HU; C, D) W: 250 HU, L: 700 HU.

**Fig. 2:**
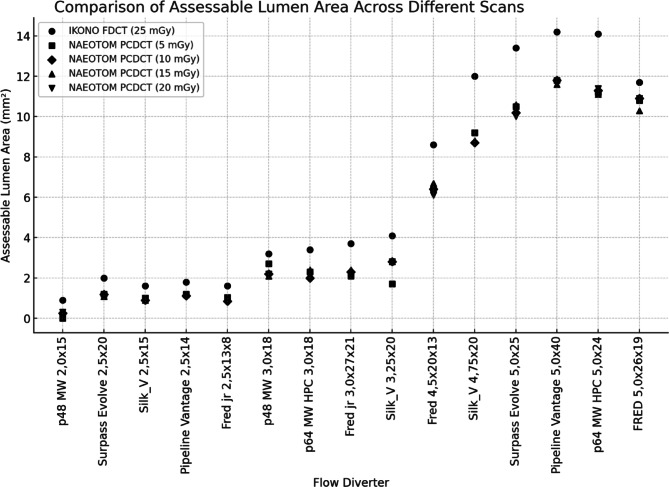
Assessable luminal area across scan modalities, radiation doses, and different flow diverter models.

The assessable lumen area varied across imaging modalities and dose levels. FP-CT provided a significantly larger assessable luminal area than PCD-CT at all dose levels (*p* < 0.05), with a median lumen area of 3.70 mm² (IQR: 1.90–11.85 mm²). PCD-CT demonstrated lower but comparable median values across dose levels: CTDI 5 (2.30 mm², IQR: 1.20–9.85 mm²), CTDI 10 (2.30 mm², IQR: 1.15–9.45 mm²), CTDI 15 (2.40 mm², IQR: 1.13–9.55 mm²), and CTDI 20 (2.20 mm², IQR: 1.14–9.60 mm²). An example is provided in Fig. 1. No significant differences were observed between PCD-CT dose levels (CTDI 5–20 mGy, ANOVA: F = 0.00007, *p* = 0.9999). Increasing CTDI from 5 to 20 mGy did not improve the assessable luminal area, indicating that higher radiation doses did not enhance stent interior visualization in PCD-CT (Fig. 2).

Across all flow diverters, PCD-CT at CTDI10 consistently underestimated the lumen area compared to FD-CT, with percentage deviations ranging from − 7% to −71%. The smallest flow diverter (2.0 mm) exhibited the largest deviation (−71%), while larger stents showed less pronounced differences. This indicates that PCD-CT at CTDI10 is less effective in assessing the lumen area, particularly for small flow diverters.

### Beam hardening artefacts in PCD-CT

**Fig. 3:**
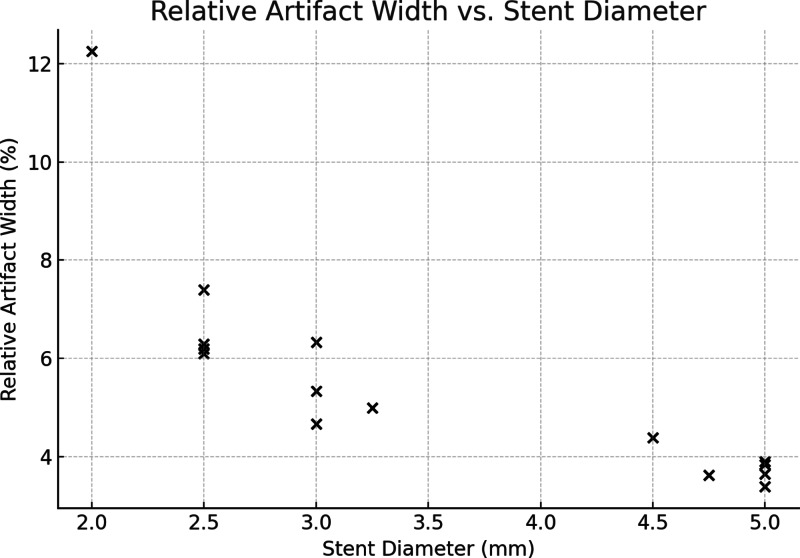
Relative beam hardening artifact width in PCD-CT imaging of flow diverters. Scatter plot showing the strong negative correlation between nominal stent diameter and relative artifact width (ρ = − 0.91, *p* < 0.001). Artifact width was defined as the distance between the contrast-filled lumen and the inner stent contour and normalized to device diameter. Smaller flow diverters exhibited proportionally greater artifact burden, consistent with reduced assessable lumen.

Quantitative analysis of the hypodense artifact adjacent to the stent lumen in PCD-CT revealed a mean relative artifact width ranging from 5.6 to 8.0% across dose levels, with no significant difference between CTDIvol 5, 10, 15, and 20 mGy (ANOVA, *p* = 0.17). However, a strong negative correlation was observed between stent diameter and relative artifact width (Spearman ρ = − 0.91, *p* < 0.001), indicating that smaller devices were disproportionately affected by artifacts (Fig. 3). These findings support the interpretation that reduced assessable lumen in PCD-CT is primarily driven by beam hardening adjacent to small-caliber flow diverters.

### CNR and SNR

**Fig. 4:**
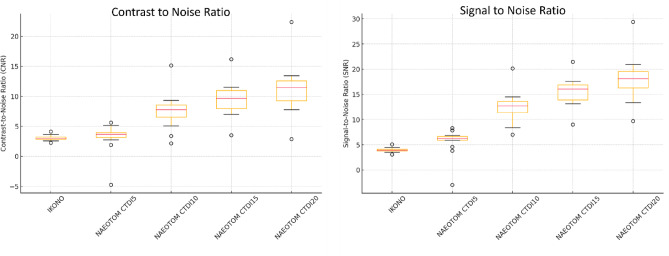
Box plots of contrast-to-noise ratio (CNR) and signal-to-noise ratio (SNR) across scan modalities and radiation dose levels.

SNR and CNR increased significantly with higher PCD-CT dose levels (*p* < 0.001), reaching the highest values at CTDI20 (SNR: 18.09, IQR: 16.29–19.59; CNR: 11.46, IQR: 9.29–12.62). FP-CT demonstrated the lowest values (SNR: 3.91, IQR: 3.77–4.10; CNR: 3.00, IQR: 2.85–3.23), whereas PCD-CT CTDI5 showed only a slight improvement over FP-CT (SNR: 6.24, IQR: 5.90–6.65; CNR: 3.68, IQR: 3.17–3.99). The largest improvement occurred from CTDI 5 to CTDI 10 (SNR + 118.1%, CNR + 137.8%, *p* < 0.001). Further increases yielded diminishing returns (CTDI 10→15: SNR + 25.5%, CNR + 28.1%; CTDI 15→20: SNR + 16.7%, CNR + 17.1%, Fig. 4). Flow diverter diameter correlated positively with SNR and CNR (Spearman ρ > 0.5, *p* < 0.05), with stronger correlations at higher doses. CTDI 10–15 mGy was identified as the optimal dose range, as post-hoc tests showed no statistically significant improvement from CTDI 15 to CTDI 20 (*p* > 0.05).

### Qualitative assessment

For subjective image quality within the stent, FP-CT achieved a median rating of 4.0 (IQR: 3.0–5.0), while PCD-CT at CTDIvol 5 had the lowest ratings (Median: 3.0, IQR: 2.0–3.0). At CTDIvol 10, 15, and 20, PCD-CT ratings improved to a median of 4.0 (IQR: 3.0–4.0), matching FP-CT but with a narrower interquartile range.

Inter-rater agreement was moderate (ICC = 0.64)^9^. ANOVA showed a significant dose effect on PCD-CT ratings (*p* < 0.001), with higher doses yielding better image quality. Tukey’s post-hoc analysis confirmed that CTDIvol 5 mGy resulted in significantly lower ratings than all other dose levels (*p* < 0.001), while CTDIvol 10–20 mGy showed no significant differences, suggesting that increasing dose beyond 10 mGy does not substantially enhance reader assessments.

**Fig. 5:**
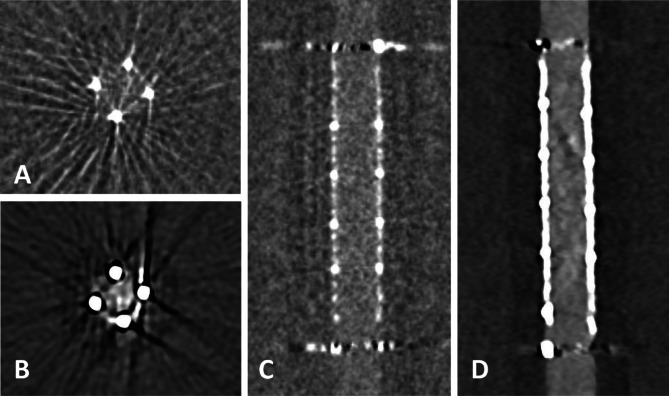
Flat panel CT (A, C) and photon-counting detector CT (CTDIvol 20 mGy; B, D) with axial and sagittal reconstructions of a FRED 4,5 × 20 × 13 mm flow diverter. Note the pronounced metal artifacts at the flow diverter ends and the limited assessability of the lumen in both modalities. Window settings were manually optimized for contrast: A, B) W: 700 HU, L: 2000 HU; C, D) W: 250 HU, L: 700 HU.

Smaller flow diverters received lower ratings, but the correlation between flow diverter diameter and image quality ratings was weak to moderate (*r* = 0.38), indicating that even small flow diverters were sufficiently assessable. FRED devices had a median rating of 3.0 (IQR: 2.0–3.0), significantly lower than all other stents (Median: 4.0, IQR: 3.0–5.0, t = −9.19, *p* < 0.001), independent of flow diverter size. No significant difference was found between FP-CT and PCD-CT for FRED devices (t = 0.25, *p* = 0.80), indicating that the imaging modality did not affect their ratings. In qualitative feedback, readers attributed the lower ratings to difficulties in assessing the stent endings due to beam hardening artifacts caused by the radiopaque marker tips. An example of the artefacts is provided in Fig. 5.

## Discussion

Our phantom study demonstrates that FP-CT and PCD-CT at CTDIvol 10 mGy provide comparable visualization of the flow diverter lumen, despite superior overall image quality with PCD-CT and a larger assessable lumen with FP-CT. Increasing the PCD-CT dose from CTDIvol 10 to 15 or 20 mGy did not significantly improve qualitative stent lumen assessment or increase the assessable lumen area. Smaller flow diverters, particularly those with a diameter < 2.5 mm, achieved better ratings on FP-CT images. However, the estimated CTDIvol for FP-CT using the manufacturer-provided protocol was approximately 27 mGy, suggesting that for medium-size to large flow diverters, PCD-CT offers a better balance between image quality and radiation dose.

The introduction of PCD-CT with its improved detector design immediately came with high hopes for all areas of radiology, including neuroradiology^1,7^. The improved resolution, until then only clinically achieved by FP-CT, combined with spectral information may provide an unprecedented image quality, concurrently bearing the potential for dose reduction. Flow diverters have gained particular attention, as follow-up evaluation of the in-stent lumen is crucial for detecting potential in-stent stenosis caused by intimal hyperplasia, which may require treatment with balloon angioplasty or extended dual antiplatelet therapy^3,17^. In our study, flow diverters with a lumen diameter of ≥ 2.5 mm achieved comparable qualitative rankings in FP-CT and PCD-CT, indicating that most flow diverters can be adequately assessed with both modalities. However, the smallest 2.0 mm lumen appeared to be better visualized with FP-CT, though this finding did not reach statistical significance, likely due to the small sample size.

Ludovichetti et al. recently evaluated phantom and clinical data of intracranial stents and flow diverters using UHR PCD-CT, demonstrating excellent device visualization after optimizing the reconstruction kernel^12^. However, no details on flow diverter size or manufacturer were provided, preventing generalizable conclusions across different devices. Their phantom data showed PCD-CT to be superior to energy-integrating detector CT, but no comparisons with DSA or FP-CT were conducted, as the primary focus was on optimizing kernel and reconstruction parameters. In our study, we used the sharp Hv72 kernel for PCD-CT reconstruction, following the optimization approach described by Ludovichetti et al. While this kernel enhances edge definition and improves stent delineation, it may also amplify high-frequency noise and beam hardening effects, potentially contributing to the reduced assessable lumen area observed in PCD-CT despite higher spatial resolution. This trade-off between spatial resolution and artifact expression should be considered when interpreting the results and selecting reconstruction parameters for clinical protocols.

In our data, PCD-CT significantly outperformed FP-CT in CNR and SNR across all CTDIvol levels. Given PCD-CT’s superior noise reduction, its advantages extend beyond stent lumen assessment to the evaluation of extravascular structures. Therefore, PCD-CT should be strongly considered when assessing larger or partially thrombosed aneurysms, peri-aneurysmal changes (e.g., edema or hemorrhage), or non-stent-related pathologies. Conversely, FP-CT remains well-suited for imaging small, high-contrast structures such as intracranial stents^12,20,24^. Due to its superior spatial resolution compared to energy-integrating detector CT, FP-CT has been shown to provide better visualization of self-expandable intracranial stents and small objects, such as cochlear implants. While its performance in detecting low-contrast structures (e.g., ischemic lesions) is reduced due to higher image noise compared to energy-integrating detector CT or PCD-CT, this limitation has minimal impact in high-contrast scenarios, such as intracranial stent and flow diverter evaluation. In our study, the higher SNR and CNR of PCD-CT did not translate into superior subjective image quality ratings. The use of a sharper reconstruction kernel, despite increased noise, appears to enhance the delineation of metallic structures and their surroundings, without interfering with in-stent assessment—a finding that has been previously reported^18,25^. Additionally, FP-CT may be less susceptible to beam hardening artifacts from metal implants due to its energy spectrum and detector characteristics. In contrast, while PCD-CT excels in noise reduction and resolution, its increased susceptibility to metal artifacts within the stent may compromise visualization, as reflected in the smaller assessable stent lumen area, potentially obscuring critical details such as linear thrombi or intimal hyperplasia. Enhancing metal artifact reduction algorithms and utilizing different energy spectrums, once spectral information becomes available for UHR mode, may help mitigate this issue. Conversely, further technological advancements, such as the ARTIS Icono’s sine spin acquisition protocol, which employs a double oblique trajectory with slight craniocaudal modulation over a 220° scan range, may reduce cone beam artifacts—particularly at the skull base—potentially improving image volume reconstruction accuracy and contrast resolution for better brain tissue visualization^19^.

The inter-reader agreement in our study was moderate, with an overall ICC of 0.64. While this suggests acceptable reliability, it falls short of the threshold typically considered strong (ICC ≥ 0.75). This level of agreement may be attributed to the overall comparable performance of both imaging modalities, which limited clear reader preference in many cases. The variability likely reflects subjective weighting of image features—some readers may have prioritized higher SNR and CNR of PCD-CT, while others valued the larger assessable lumen area seen with FP-CT. This suggests that reader-specific preferences influence image interpretation when diagnostic performance is closely matched. Further standardization or reader training may help reduce such variability in clinical decision-making, since this level of variability could affect clinical decision-making in borderline cases.

In our study, both FRED and FRED Jr. flow diverters exhibited increased image noise at both ends, making stent entrance evaluation challenging across both modalities. This is likely due to the unique design of the FRED flow diverter^15^. Unlike other flow diverters, FRED features a dual-layer structure, consisting of an inner flow diverter within an outer stent-like framework, facilitating deployment in tortuous anatomies. An interwoven double-helix of radiopaque tantalum strands connects the inner and outer layers, enhancing visibility along the entire dual-layered section. The flared ends, marked by four radiopaque tips, improve fluoroscopic visibility, particularly at the skull base, but introduce challenges in follow-up imaging due to significant artifacts at the stent entrances. Subgroup analysis of our data did not support the hypothesis that FP-CT is superior to PCD-CT for FRED devices.

Limitation of this study is mainly the small sample size and the in vitro evaluation, especially in terms of generalizability to real-world clinical settings, such as patient movement, varying contrast levels, and diverse anatomical structures such as the skull base. Especially tortuous anatomies, vessel wall interactions, deformation, or apposition variability seen in vivo are not reflected in our model. Furthermore, DSA remains the clinical gold standard for evaluating flow diverters. Without a direct DSA correlation, the diagnostic value of FP-CT and PCD-CT for detecting in-stent complications, such as thrombus formation or intimal hyperplasia, remains unverified. Moreover, the true in-stent lumen could not be verified against histological analysis or high-resolution ex vivo imaging, limiting absolute accuracy validation. Future studies should concentrate on validating our findings in vivo and optimizing image protocols for both modalities to improve the post interventional follow-up of flow diverters in every day clinical setting over the whole spectrum of available flow diverters and intracranial stents.

## Conclusion

Our study demonstrated that while FP-CT provided superior visualization of the flow diverter lumen, PCD-CT achieved comparable subjective assessability ratings when evaluated by experienced readers. Despite higher SNR and CNR in PCD-CT, these advantages did not translate into better stent lumen visualization, likely due to increased metal artifacts affecting the assessable area. Among the PCD-CT dose levels, CTDIvol 10 mGy provided the best balance between image quality and radiation dose, as increasing the dose further did not significantly improve lumen assessment. Smaller flow diverters (≤ 2.5 mm diameter) showed a tendency toward better visualization with FP-CT, although the sample size was limited and the difference was not statistically significant.

## Methods

### Phantom

A standardized acrylic glass CTDI head phantom (T40017, PTW, Freiburg, Germany) with a diameter of 16 cm was used. The phantom contains five holes—one in the center and four symmetrically distributed around the periphery. The peripheral holes were filled with acrylic dummy plugs, while the central hole was used to accommodate the vessel model.

The vessel models were designed using Fusion360 (Autodesk Inc., San Francisco, CA, United State) and fabricated with a ProJet MJP 2500 multijet 3D printer (3D Systems, Rock Hill, SC, United States) to accommodate various flow diverter designs and appropriate vessel sizes. The material used was 3D Systems VisiJet M2S-HT90. This printer was chosen for its high dimensional accuracy, with a maximum deviation of 0.1016 mm over 25.4 mm. Additionally, the surface finish is not affected by the waxy support material (3D Systems VisiJet M2 SUP), which melts away cleanly without residue after printing. M2S-HT90 is a UV-curing plastic that is transparent, dimensionally stable, resistant to high temperatures, and has low water absorption, making it nearly chemically inert. The outer diameter of each model was aligned with the inner surface of the head phantom. To prevent potential air pockets, ultrasound gel was applied before insertion. The inner lumen of the vessel models corresponded to the size of the respective flow diverters. The density of the tube was ca. 100 HU. 15 different flow diverters, varying in design and size, were provided by the manufacturers and inserted under fluoroscopy into the appropriate vessel models.

The tube was then filled with a solution of 0.9% saline and contrast agent (Imeron 300, Bracco Imaging, Italy) with a density of 300 HU, simulating typical vessel contrast in head and neck CT angiography, using a scan with 120 kVp. The phantom was placed parallel to the z-axis of the CT scanner or flat detector system and centered at the isocenter, with the plastic tube containing the flow diverter positioned outside the isocenter.

*Flow Diverter*.

15 flow diverters with different sizes, provided by different manufactures, were examined, reflecting the broad variety of options of flow diverters on the market. The companies had no influence in the study design and the formulation of the paper. Details to size, length and manufacturer are presented in Table 1.

**Table 1:** Flow diverter properties.

Denomination	Manufacturer	Diameter (in mm)	Length (in mm)	Material
**p48 MW 2**,**0 × 15**	phenox GmbH	2.0	15	Nitinol/Platinum
**Surpass Evolve 2**,**5 × 20**	Stryker Corp.	2.5	20	Cobalt-Chromium/ Platinum-Tungsten
**Silk_V 2**,**5 × 15**	Balt SAS	2.5	15	Nitinol/Platinum
**Pipeline Vantage 2**,**5 × 14**	Medtronic plc	2.5	14	Cobalt-Chromium/Platinum
**FRED Jr. 2**,**5 × 13 × 8**	MicroVention, Inc.	2.5	13	Nitinol/Tantalum
**p48 MW 3**,**0 × 18**	phenox GmbH	3.0	18	Nitinol/Platinum
**p64 MW HPC 3**,**0 × 18**	phenox GmbH	3.0	18	Nitinol/Platinum
**FRED Jr. 3**,**0 × 27 × 21**	MicroVention, Inc.	3.0	27	Nitinol/Tantalum
**Silk_V 3**,**25 × 20**	Balt SAS	3.25	20	Nitinol/Platinum
**FRED 4**,**5 × 20 × 13**	MicroVention, Inc.	4.5	20	Nitinol/Tantalum
**Silk_V 4**,**75 × 20**	Balt SAS	4.75	20	Nitinol/Platinum
**Surpass Evolve 5**,**0 × 25**	Stryker Corp.	5.0	25	Cobalt-Chromium/ Platinum-Tungsten
**Pipeline Vantage 5**,**0 × 40**	Medtronic plc	5.0	40	Cobalt-Chromium/Platinum
**p64 MW HPC 5**,**0 × 24**	phenox GmbH	5.0	24	Nitinol/Platinum
**FRED 5**,**0 × 26 × 19**	MicroVention, Inc.	5.0	26	Nitinol/Tantalum

### *Acquisition protocols and image reconstruction*

#### PCD-CT

Images were acquired using a novel PCD-CT system (NAEOTOM Alpha; Siemens Healthineers, Erlangen, Germany) with standard clinical protocols. Scanning was performed in ultra-high resolution (UHR) mode at a tube voltage of 120 kVp. To achieve target dose levels, defined by volume CT dose index (CTDIvol) values of 5 mGy, 10 mGy, 15 mGy, and 20 mGy, the tube current–time product (mAs) was manually adjusted. The selected CTDIvol levels reflect a clinically relevant dose range, with 5 mGy representing the lowest clinically realistic protocol and 20 mGy being the maximum dose allowed by the scanner software for the ultra-high-resolution mode. Each device was scanned once with each dose level. The smallest available field of view (50 mm × 50 mm in the X and Y axes) was used, with a reconstructed minimum slice thickness of 0.2 mm in the Z-axis. The matrix size was set to 1024 × 1024, resulting in an approximate voxel size of 0.05 × 0.05 × 0.2 mm. In UHR mode, which provides the highest spatial resolution in PCD-CT, spectral information is not available. Based on prior studies on intracranial stents, flow diverters, and cardiac stents using PCD-CT^12,14^, the Hv72 kernel was selected for optimal visualization of the stent and contrast-filled lumen. A third-level iterative reconstruction algorithm was applied.

#### Flat panel CT

Images were acquired on a state-of-the-art angiographic system (ARTIS Icono; Siemens Healthineers, Erlangen, Germany). A protocol for intravenous contrast media application was employed for the FP-CT. The predefined tube voltage was 90 kVp, and the tube current was 390 mAs, resulting in the generation of 248 images over a four-second period with an average DAP of 2139.8µGym^2^. Subsequently, the images were reconstructed using the smallest field of view possible, measuring 74 × 74 mm, and the largest available matrix size in the X and Y axes, which was 512 × 512 using the convolution kernel HU/sharp. Reconstructed slice thickness was 0.14 mm. The voxel size of 0.14 × 0.14 × 0.14 was isometric. Each device was scanned once without re-insertion, to avoid introducing variability from repositioning.

FP-CT do not by default provide CTDIvol values. However, for comparison, we conducted additional measurements on our head phantom to estimate the CTDIvol of the FP-CT protocol according to standard CT measurement protocols. We utilized a calibrated dosimetry system, the Nomex dosimeter from PTW (Freiburg, Germany) with an associated 100 mm CT ionization chamber and the setup for CTDI head phantoms. The isocentrically placed head phantom required an average total dose of approximately 25 mGy (measured as CTDIvol) for the FP-CT protocol. However, it is important to note that standard CTDI measurement protocols cannot be fully applied to FP-CT systems due to inherent differences in their acquisition methods, particularly the lack of a full 360-degree rotation during image acquisition. Therefore, this measurement can only provide a dose approximation for the FP-CT.

#### Image analysis

Three board-certified interventional neuroradiologists with extensive experience in evaluating flow diverter implantation during interventions and follow-up imaging across all available modalities (AB, LB, and FJS) conducted the qualitative image analysis. Lumen assessability was evaluated qualitatively using volumetric data in all three imaging planes and rated on a 5-point Likert scale: 1 = extensive artifacts, stent lumen not assessable; 2 = barely visible stent lumen with significant artifacts; 3 = sufficiently visible stent lumen with moderate artifacts; 4 = assessable stent lumen with mild artifacts; 5 = minimal artifacts, with the majority of the stent lumen clearly assessable.

Quantitative image analysis was performed by an experienced neuroradiologist (CJM) to ensure consistency and to minimize variability in ROI placement. Regions of interest (ROIs) were placed in the contrast-filled stent lumen and the acrylic phantom on the same image slice. For each scan, one ROI was manually placed at the central segment of the stent lumen in a plane orthogonal to the flow diverter, resulting in one measurement per device per dose level. ROI size was maximized while carefully avoiding adjacent structures, particularly stent meshes and artifacts. ROI size ranged from 0.3 mm² for the smallest to 11 mm² for the largest flow diverter, while avoiding stent struts; in the acrylic phantom, fixed ROIs of approximately 100 mm² were used to reduce statistical noise and improve measurement stability. Signal-to-noise ratio (SNR) was calculated as the ratio of the mean Hounsfield Unit (HU) value within the flow diverter ROI to the standard deviation of the phantom. Contrast-to-noise ratio (CNR) was determined by dividing the absolute difference between the mean HU values of the stent lumen and the phantom by the standard deviation of the phantom. The area of the contrast-filled visible stent lumen was measured centrally within the stent in a plane strictly orthogonal to the flow diverter.

Beam hardening artifacts beneath the stent struts in PCD-CT as depicted in Fig. 1 were measured using the method proposed by Adolf et al.^2^. Artefact width was defined as the linear distance (in mm) between the edge of the contrast-filled lumen and the inner contour of the visible stent wall, measured in axial orientation. To account for differences in device size, we calculated the relative artifact width according to the following formula:

$$\:relative\:artifact\:width\:\left(\%\right)=\frac{artifact\:width\:\left(mm\right)}{nominal\:stent\:diameter\:\left(mm\right)}\:x\:100$$.

##### Statistical analysis

Statistical analysis was performed using Python 3.10, employing the SciPy, statsmodels, and pandas libraries for data processing and statistical computations. For quantitative analysis, assessable lumen area was compared using paired t-tests, while CNR and SNR were analyzed across imaging modalities and dose levels using one-way ANOVA with Tukey’s post-hoc test. Pearson correlation was used to assess the relationship between flow diverter diameter and quantitative metrics. For qualitative analysis, interreader variability was evaluated using the Intraclass Correlation Coefficient (ICC), Fleiss’ Kappa, and Bland-Altman analysis to detect systematic biases. The effect of dose level on subjective ratings was analyzed using one-way ANOVA with Tukey’s post-hoc test, and Welch’s t-test was applied to compare ratings of FRED flow diverters with other models. Dose dependence of the beam hardening artifact were analyzed using a one-way ANOVA across four CTDIvol​ levels (5, 10, 15, 20 mGy) with relative artifact width (%) as the dependent variable. A Spearman rank correlation was calculated between nominal stent diameter (mm) and mean relative artifact width (%). A significance threshold of *p* < 0.05 was applied to all statistical tests.

## Data Availability

Data availabilityThe datasets used and/or analyzed during the current study are available from the corresponding author onreasonable request.

## Abbreviations

FP: CT–Flat–Panel Computed Tomography
PCD: CT–Photon–Counting Detector Computed Tomography
UHR: Ultra–High Resolution
ROI: Region of Interest
